# Relationship between lower limb neuromuscular and functional performance tests and sprint and agility in female volleyball players: a cross-sectional study

**DOI:** 10.3389/fphys.2026.1827693

**Published:** 2026-04-20

**Authors:** Soner Akgün, Müjde Atıcı, Akan Bayrakdar, Esra Korkmaz Salkılıç, Berna Anıl, Enes Akdemir, Dilara Kumru, Kenan Şebin, Yener Aksoy, Ali Kerim Yılmaz

**Affiliations:** 1 Faculty of Sports Sciences, Artvin Çoruh University, Artvin, Türkiye; 2 Faculty of Sports Sciences, Kahramanmaraş Sütçü İmam University, Kahramanmaraş, Türkiye; 3 Faculty of Sports Sciences, Alanya Alaaddin Keykubat University, Antalya, Türkiye; 4 Faculty of Yaşar Doğu Sports Sciences, Ondokuz Mayıs University, Samsun, Türkiye; 5 Turkish Curling Federation, Ankara, Türkiye

**Keywords:** agility, functional performance tests, lower limb, sprint, volleyball

## Abstract

**Background:**

The aim of the study was to examine the relationship between vertical and horizontal neuromuscular and functional jump tests applied to the lower extremities of female volleyball players and their agility and sprint performance.

**Methods:**

22 women aged 18-25 (average age 20.18, average height 173 cm, average weight 58.36 kg, and average body mass index (BMI) 19.63 kg/m²) voluntarily participated in the study. The sprint test (10m-30m) and 505 Agility tests were used to determine participants’ sprint and agility skills, respectively. Additionally, the countermovement jump (CMJ), drop jump (DJ), and five different single-leg hop tests (SLHTs)—single-leg hop for distance (SH), triple hop for distance (TH), crossover hop for distance (CH), medial side triple hop (MSTH), and medial rotation hop (MRH)—were used to determine lower-extremity neuromuscular and functional jump performance.

**Results:**

The results of the study revealed a moderate to high negative correlation between CMJ and DJ and sprint tests (p<0.05). A negative and moderately significant correlation was observed between CMJ and agility (p<0.05), but the correlation between DJ and agility was not significant (p>0.05). A moderate to high negative correlation was found between SLHTs and sprint and agility (p<0.05). Also, there was no significant difference between participants’ dominant (D) and non-dominant (ND) side SLHT jump distances (p>0.05), and limb symmetry indices (LSI) were within the normal range.

**Conclusions:**

In conclusion, it was determined that lower extremity neuromuscular and functional vertical and horizontal (neuromuscular and functional) strength plays a critical role in linear acceleration and change of direction performance in female volleyball players. It has been suggested that not only vertical jump tests but also horizontal functional jump tests such as SLHTs should be more comprehensively investigated in relation to sprinting and agility in female volleyball players.

## 1 Introduction

Volleyball is a dynamic sport characterized by high-intensity, explosive movements that involve frequent repetition of multi-directional actions such as acceleration, deceleration, changes in direction, and jumping, performed in short intervals (Lima et al., 2023; Rebelo et al., 2025). These short bursts of activity are usually followed by brief rest/recovery periods of 10–20 seconds (Yücel, 2026). Sudden stops, re-accelerations, and repeated jumps in volleyball players require high neuromuscular control, dynamic balance, and reactive strength in the lower extremity muscle-tendon structures (García-de-Alcaraz et al., 2020; Popowczak et al., 2021; Wang et al., 2025). Considering the importance of lower extremity functions in volleyball, evaluating these functions correctly and comprehensively is crucial for both improving performance and reducing the risk of injury (Velarde-Sotres et al., 2025; Wang et al., 2025; Sitti and Köroğlu, 2026).

There are various test procedures that enable the measurement of the neuromuscular functions and functional strength of the lower extremities (Velarde-Sotres et al., 2025). Some of the most commonly used tests today are the Countermovement Jump (CMJ), Drop Jump (DJ), and Single Leg Hop Tests (SLHT) (Xu et al., 2023; van Melick et al., 2024). CMJ evaluates the coordination of eccentric and concentric contractions of the muscle-tendon unit, while DJ measures reactive strength production and the effectiveness of the stretch-shortening cycle (SSC). SLHTs aim to assess single-leg balance, strength, and function while also helping us evaluate the relationship between bilateral symmetry ratios and disability (Harper et al., 2020; Keiner et al., 2024; Sohrabi and Naderi, 2025). Also, parameters obtained through explosive power, such as CMJ, DJ, and SLHT, are closely related to athletic skills that require explosive power and rapid directional changes, such as sprinting and agility (Gisladottir et al., 2024; Xu et al., 2024a).

In volleyball, where jumping performance is a key factor, fundamental skills such as attack, block, and defense, when combined with short sprints and the ability to change direction suddenly, enable players to strike the ball accurately and position themselves effectively (Silva et al., 2019; Pleša et al., 2021; Pawlik and Mroczek, 2023). The high repetition rate of these movements, which require instant decision-making, may also increase the risk of injury by increasing neuromuscular load on the lower extremities (de Leeuw et al., 2022; Zhao et al., 2024). Hence, regularly monitoring jump performance and agility and speed parameters in volleyball players is crucial for both improving performance and assessing injury risks.

Studies have reported that high CMJ and DJ scores in volleyball players are associated with reduced sprint times and better performance in agility tests (Barnes et al., 2007; Schaun et al., 2013; Gulati et al., 2021; Atabaş et al., 2026). These findings clearly demonstrate that explosive strength and the effectiveness of the stretch-shortening cycle in volleyball support short-distance acceleration and change-of-direction skills. However, studies examining the relationship between SLHT performance in volleyball players and agility movements requiring single-leg balance and strength, as well as sprint times, are still limited (Sohrabi and Naderi, 2025).

When all these results are evaluated in light of the literature findings, our current study aims to reveal the relationship between lower extremity neuromuscular and functional outcomes assessed both vertically (CMJ, DJ) and horizontally (SLHT) in the same group of volleyball players and their agility and sprinting performance. The study hypothesized that there would be significant correlations between sprinting and agility based on both vertical and horizontal lower extremity jump tests.

## 2 Materials and methods

### 2.1 Study design

**Figure 1:**
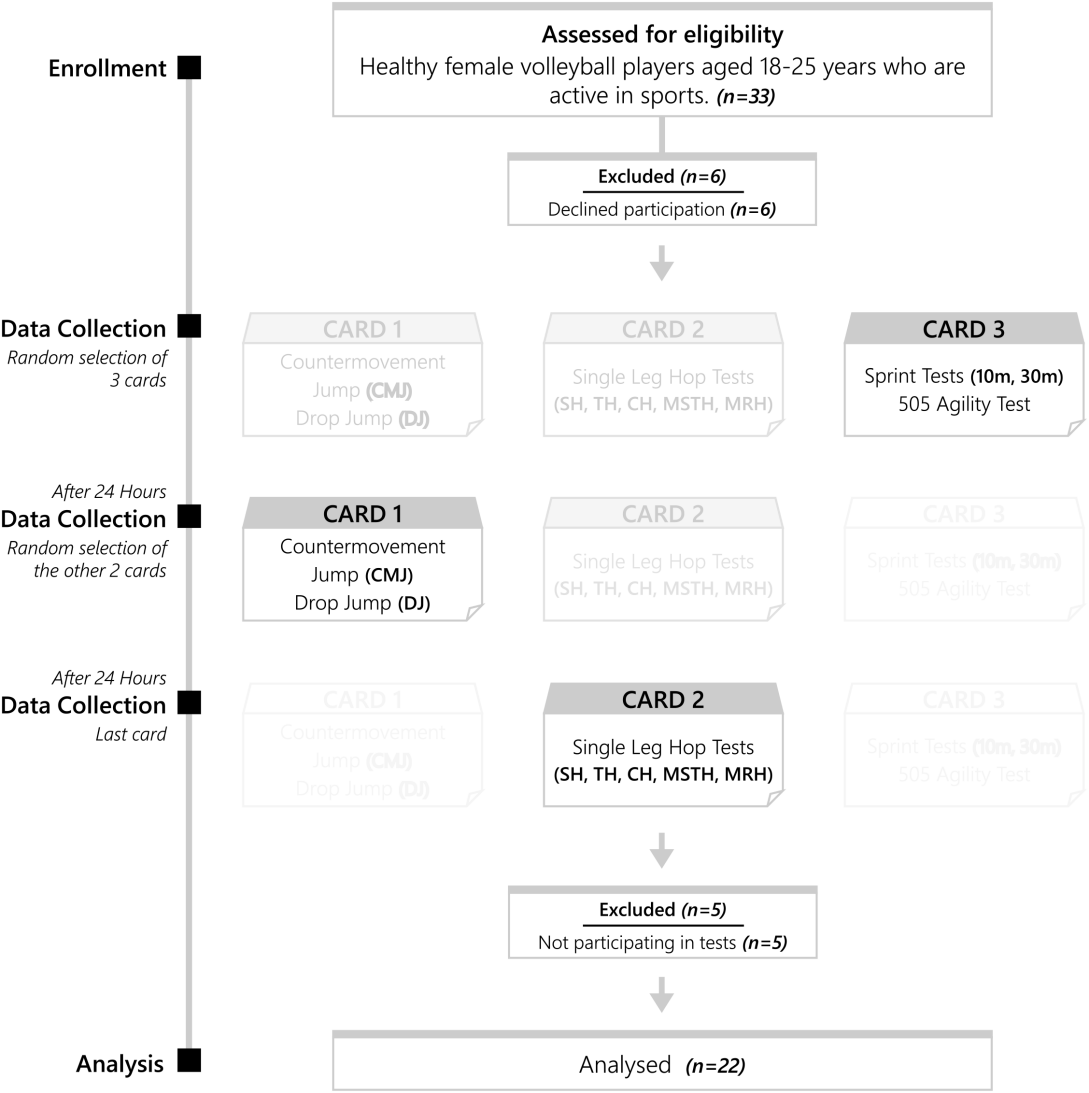
Flowchart.

This study has a cross-sectional study design that includes randomized test protocols. The participants visited the laboratory twice, including the introductory session (**Figure 1**). During the introductory session, participants were provided with information about the study, and a practical demonstration was conducted regarding the tests they would be performing during the study period. The anthropometric measurements specified in the measurement method were also performed during this session. During the second visit, participants were asked to select randomized application cards, and measurements were taken accordingly. The procedures on the randomized application cards are listed below:

1st Card: Countermovement Jump (CMJ) and Drop Jump (DJ); 2nd Card: Single Leg Hop Tests [single leg hop for distance (SH), triple hop for distance (TH), crossover hop for distance (CH), medial side triple hop (MSTH), and medial rotation hop (MRH)]; 3rd Card: 10 m. and 30 m. Sprint Test, 505 Agility Test.

The participants rested for 24 hours after each session. The SH, TH, CH, MSTH, and MRH tests were applied separately to both the right and left sides. CMJ and DJ tests were performed bilaterally. In the CMJ test, two different protocols were applied: one with arm swing (CMJ AS) and one without arm swing (CMJ WAS), while in the DJ test, two different box heights [20 cm (DJ 20) and 40 cm (DJ 40)] were used. All performance assessments were conducted using standardized protocols, the validity and reliability of which have been extensively documented in prior research (Booher et al., 1993; Dingenen et al., 2019; de Pedro-Múñez et al., 2025). The rest period between each test was kept to at least 5 minutes. Despite the rest periods, the time for participants who were not ready for the next test was extended up to twice as long. Immediately before the tests, participants underwent a 10-minute general warm-up protocol (dynamic stretching, mobility exercises) for the lower extremity muscle groups. Participants were instructed not to engage in physical activity or exercise programs for at least 48 hours prior to the measurements and to avoid stimulants (such as caffeine) during this period. Measurement sessions organized for all individuals during the study were conducted at the same time of day (1:00 PM to 3:00 PM) and under the same environmental conditions (a temperature between 19-22 °C and a humidity level between 50-60%). Each session ended with a 10-minute cool-down protocol (static stretching and breathing exercises). The study was conducted in accordance with the ethical principles outlined in the Helsinki Declaration. Ethics committee approval was obtained from Alanya Alaaddin Keykubat University Non-invasive Clinical Research Ethics Committee (protocol no: 2025/07).

### 2.2 Participants

The study was conducted with the voluntary participation of 22 female volleyball players aged 18-25 (**Table 1**). The sample size for the study was determined using G×Power analysis (version 3.1.9.6, Germany) (d=0.8; α=0.05; 1-β=0.8), and the minimum number of participants was determined to be 18. Inclusion criteria for the study were agreeing to participate voluntarily in the study, being a female volleyball player aged 18-25, having participated in regular volleyball training for at least 5 years, having no history of serious sports injuries in the last 6 months, being healthy, and having no chronic conditions. Participants who had a history of serious sports injury within the last 6 months, experienced any serious injury or discomfort during the study, wished to withdraw from the study, or did not meet the other inclusion criteria were excluded from the study. All participants have actively participated in regular training programs consisting of 3 training sessions per week over the past 5 years, with each session lasting an average of 90 minutes. An informed consent form was obtained from participants who met the inclusion criteria prior to the study.

**Table 1:** Descriptive data.

Variables	Mean	SD	Min	Max
Age (year)	20.18	1.79	18.00	25.00
Height (cm)	173	1.04	165	178
Weight (kg)	58.36	3.90	54.00	70.00
BMI (kg/m²)	19.63	1.35	17.96	22.86
Training Age (year)	9.59	1.10	8.00	15.00

SD, standard deviation; Min, minimum; Max, maximum; BMI, body mass index.

The participants’ descriptive data are presented in **Table 1**.

### 2.3 Evaluation tools

#### 2.3.1 Anthropometric measurements

Participants’ body weights were measured with an accuracy of 0.1 kg using a body composition analyzer (Jawon Body Composition Analyzer Model X-Scanplus II, Seoul, Korea). Height measurements were taken using a stadiometer (Holtain Ltd., Crymych, UK) with an accuracy of 0.1 cm. Both measurements were performed barefoot and in an anatomical stance (Lohman et al., 1988). Body mass index (BMI) was calculated using the formula ‘body weight (kg)/height (m)² ‘ from the obtained body weight and height data.

#### 2.3.2 505 agility test

The 505 agility test times of the participants were determined using a photocell (Seven, SE-165 Photocell Stopwatch, İstanbul, Türkiye). Distances of 0 m, 10 m, and 15 m were determined on a flat surface. A photocell was placed in the 10 m section of the test area. The participants ran 10 meters from the starting point (0 m) and started the timer by passing through the photocell timing gate. The participants continued running, reached the 15 m line in the test area, then made a 180° turn and passed the same photocell again to stop the time. The participants performed the test twice, and the best time achieved was recorded in seconds (Ja and Draper, 1985).

#### 2.3.3 Sprint tests (10m and 30m)

The participants’ 10- and 30-meter sprint times were determined using a photocell (Seven, SE-165 Photocell Stopwatch, İstanbul, Türkiye). The test was conducted on a flat and hard running track. Participants performed a 10-minute general warm-up protocol (light jogging, mobilization, and a few short sprints) immediately before the application session. When the participants were ready, they took their places behind the starting line for the 30m speed test, and at this point their toes did not touch the line. After the ready command, participants began running whenever they wanted. After the start, the measurement began when passing through the first photocell gate, and it automatically ended when passing through the last photocell gate located at the end of the 30-meter distance. The same protocol was applied for the 10 m sprint test, and participants were asked to perform two repetitions for each test. Full rest periods (3–5 minutes) were given between repetitions. Participants’ best performance during the analysis was recorded (Lockie et al., 2003; Brown and Ferrigno, 2005).

#### 2.3.4 Single-leg hop tests

The SLHTs were performed on a 6m-long and 15 cm-wide measuring tape placed on a flat surface. The participants placed their toes on the starting point of the measuring device and waited in a balanced position on one leg, ready to perform the test whenever they wanted. In the tests, participants were required to maintain balance on one leg for 3 seconds after landing on that leg as a criterion for success. Arm swing was unrestricted during the test. SLHTs consist of 5 different tests. In the SH test, participants performed a single jump while standing on one foot at the starting point. In the TH test, participants stood on one foot and performed three consecutive jumps forward along the measuring tape. In the CH test, participants stood on one foot at the starting line boundary and, when ready, performed three consecutive jumps by crossing over to the opposite leg from the one used for the first jump. In the MSTH test, participants placed the medial side of their feet on the boundary of the starting line and then performed three consecutive jumps in the medial direction. In the MRH test, participants were asked to place the medial part of their feet at the boundary of the starting point. When the participants were ready, they performed a medial jump as far as possible by performing a right-angled medial rotation. Three trials were conducted with the participants before all main tests. In all jumps that met the success criteria in the main tests, the distance between the heel height of the participants and the starting point was recorded in centimeters (Munro and Herrington, 2011; Dingenen et al., 2019; Peebles et al., 2019).

#### 2.3.5 Counter movement jump test

Participants’ CMJ jump heights were measured using a dual force plates (ForceDecks, FDLite V.2, VALD, Brisbane, Australia). The test was administered in two ways: with arm swing (CMJ AS) and without arm swing (CMJ WAS). For both tests, participants stood upright with their feet hip- and shoulder-width apart, then quickly bent their knees and hips to squat down and performed a vertical jump with maximum strength. During the jump, the legs were kept straight and the body was in an upright position. The athletes bent their knees softly as they landed on the ground and were deemed successful when they maintained their balance. In the test where arm swing was not permitted, participants held their arms at waist level, while in the test where swinging was not restricted, participants were free to swing their arms forward and/or upward (Markovic et al., 2004).

#### 2.3.6 Drop jump test

Participants’ DJ jump heights were determined using a dual force plates (ForceDecks, FDLite V.2, VALD, Brisbane, Australia). The subject performed dynamic warm-up and joint mobility exercises before starting the test. After the warm-up session, the participants placed her hands on her hips, stood up straight, and stepped onto the raised box behind the power platform. Without accelerating upward, the participants performed a free fall onto the platform by taking a step forward, landing with both feet simultaneously. Immediately upon contact (with minimal contact time), the participant jumped vertically to maximum height; then, the participants landed softly on the platform, maintained balance, and remained motionless for 2–3 seconds. The test was administered twice to participants with full rest intervals, and the best jump height was recorded in centimeters (Carlock et al., 2004).

### 2.4 Statistical analysis

The SPSS software package (SPSS version 25.0, IBM) was used for statistical analysis. Mean values and standard deviation were used for descriptive data. The normality and equality of variance of the data were assessed using the Shapiro-Wilk, Q-Q plot, and Levene tests, respectively, and the data were found to be normally distributed. The paired samples t-test was used for pairwise comparisons, while the Pearson correlation test was used for correlational analysis. In correlation analyses, correlations above 0.30 were considered weak, 0.3–0.6 moderate, 0.6–0.8 strong, and 0.8–1.0 perfect (Akoglu, 2018). In the comparison of matched groups, effect sizes were calculated according to Cohen’s d effect size (M2 - M1) / pooled SD). As per this formula, d < 0.2 was defined as a weak effect size, d = 0.5 as a moderate effect size, and d > 0.8 as a strong effect size. The significance level was determined as p < 0.05 in all tests.

## 3 Results

**Figure 2:**
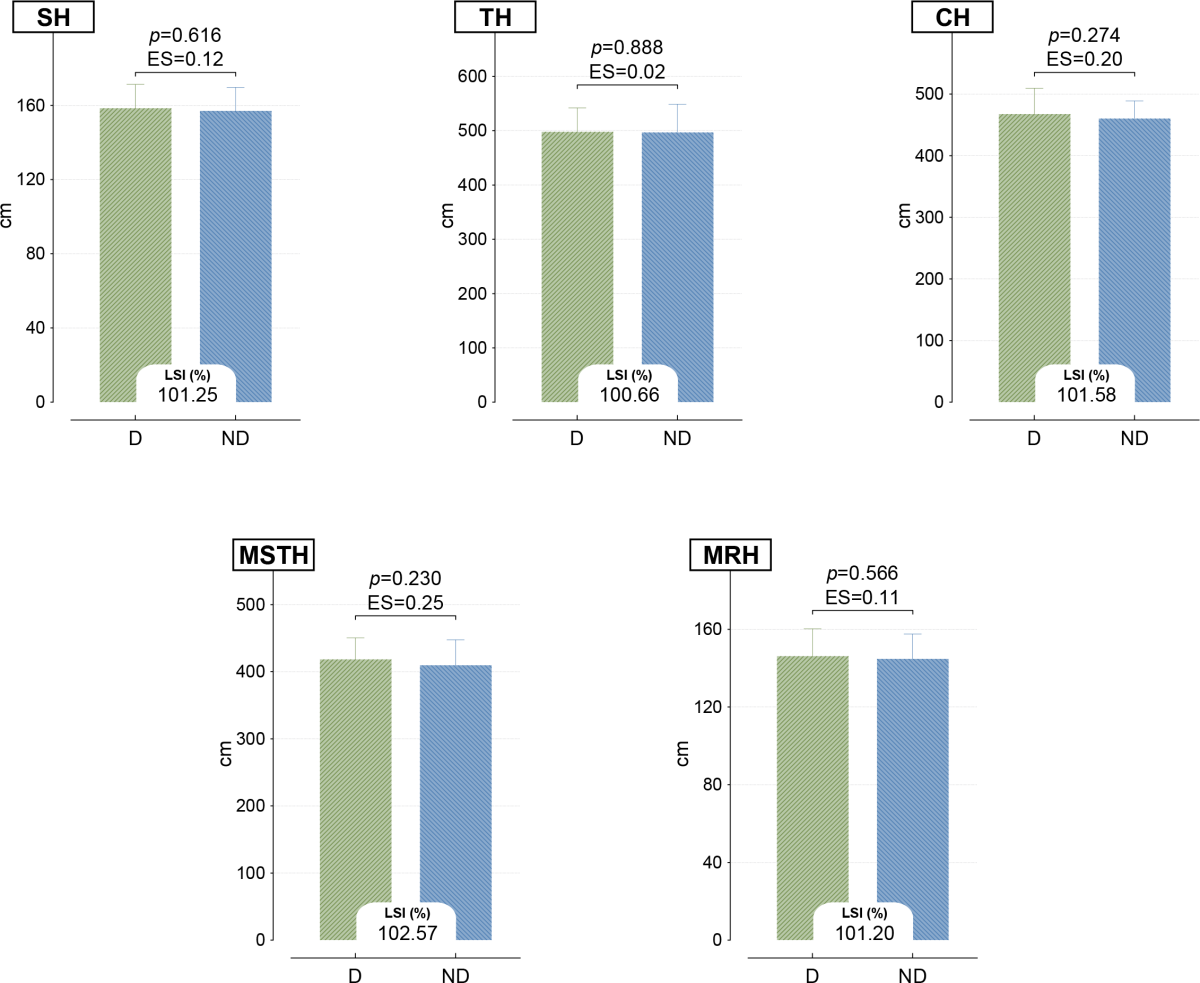
Comparison of dominant and non-dominant SLHTs. SH, single leg hop for distance; TH, triple leg hop for distance; CH, crossover hop for distance; MSTH, medial side triple leg hop for distance; MRH, medial rotation hop for distance.

**Figure 2** shows the SLHT distances on the D and ND sides. When looking at the findings, the results between the parties were found to be similar for all SH, CH, TH, MSTH, and MRH parameters (p > 0.05). Furthermore, it was observed that the obtained LSI ratios were within normal ranges (± 10-15%).

**Figure 3:**
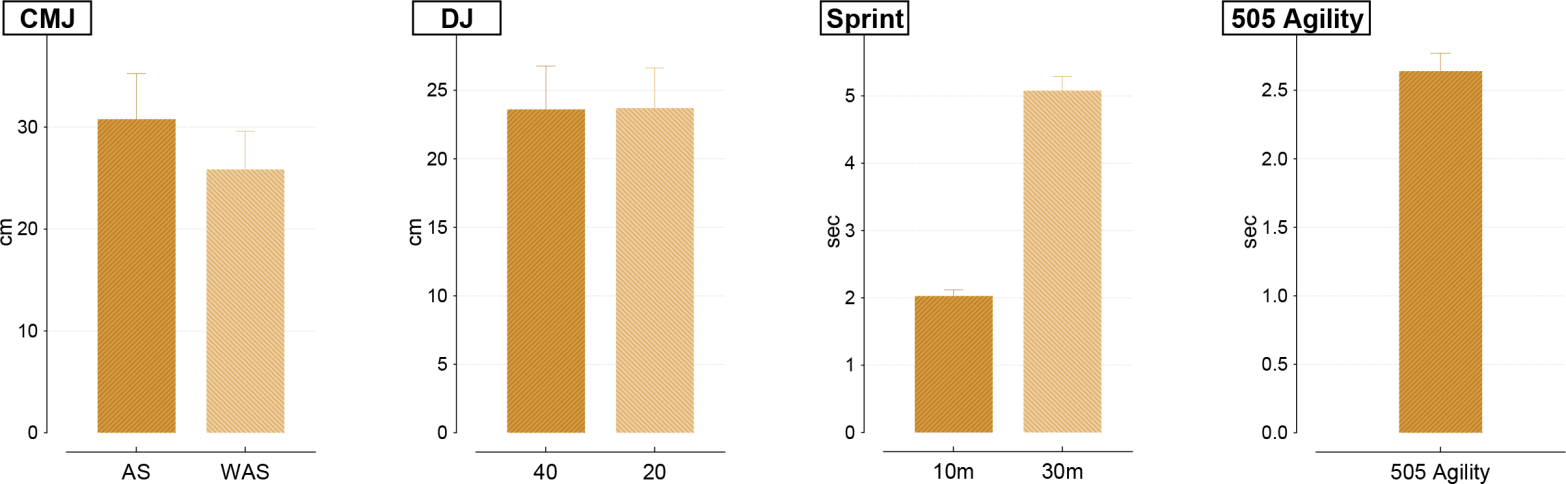
Vertical jump, sprint, and agility test results of the participants. CMJ, countermovement jump test; DJ, drop jump; AS, with arm swing; WAS, without arm swing.

**Figure 3** presents the descriptive data of the participants’ CMJ AS, CMJ WAS, DJ 40, DJ 20, 10 m sprint, 30 m sprint, and 505 Agility tests.

**Figure 4:**
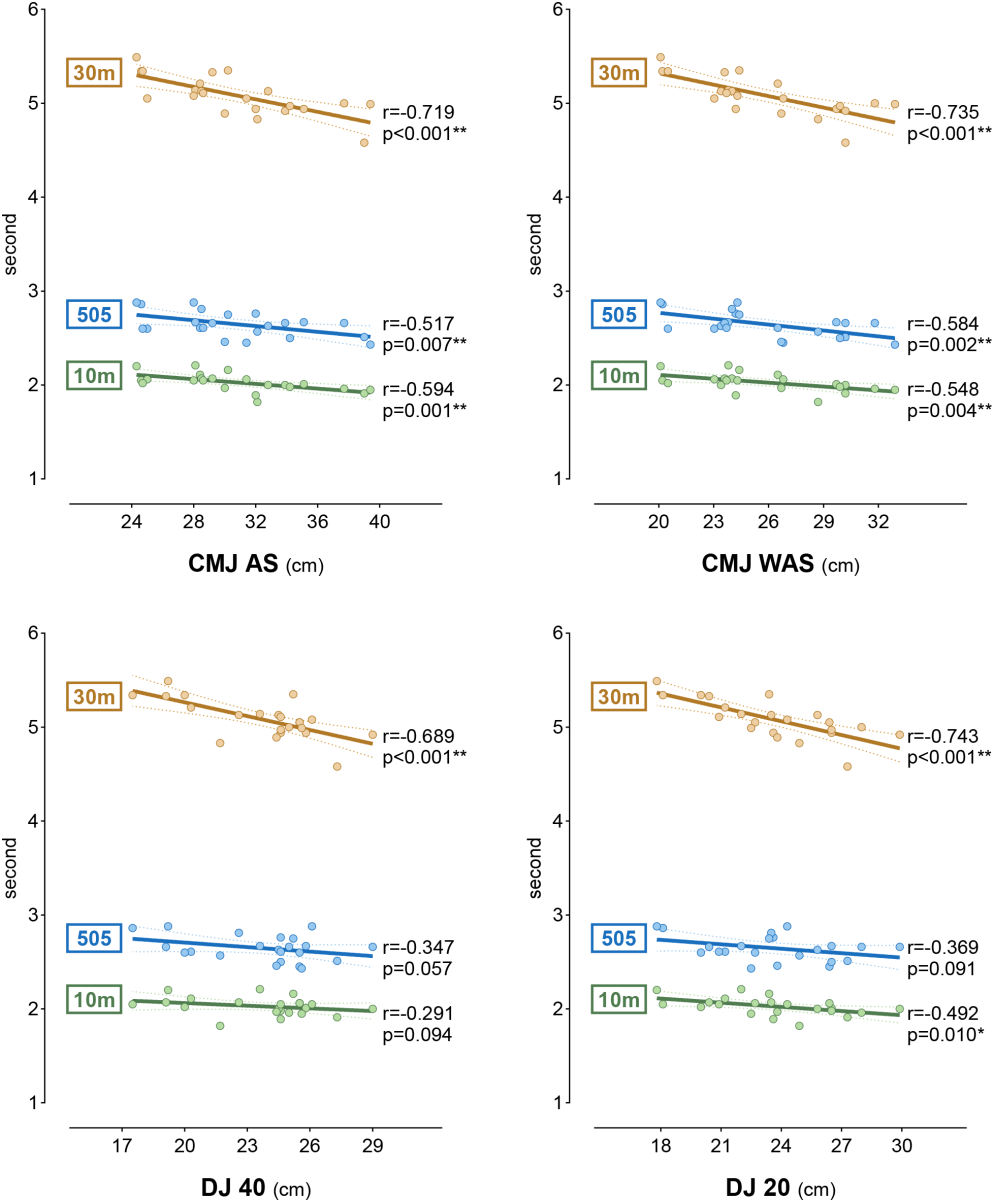
The relationship between jump tests and sprint and agility tests. *p<0.05; **p<0.01; r, results of Pearson correlation; DJ, drop jump; CMJ WAS, without arm swing countermovement jump test; CMJ AS, with arm swing countermovement jump test.

The correlation between participants’ sprint, agility, and jump performance is presented in **Figure 4**. When the results were evaluated, there was a moderate negative correlation between the 10 m sprint and CMJ AS, CMJ WAS, and DJ 20 (r = -0.594, -0.548, -0.492); a high negative correlation between the 30 m sprint and CMJ AS, CMJ WAS, DJ 40, and DJ 20 (r = -0.719, -0.735, -0.689, -0.743); and a moderate negative correlation between the 505 agility and CMJ AS and CMJ WAS (r = -0.517, -0.584) (p < 0.05, p < 0.01).

**Figure 5:**
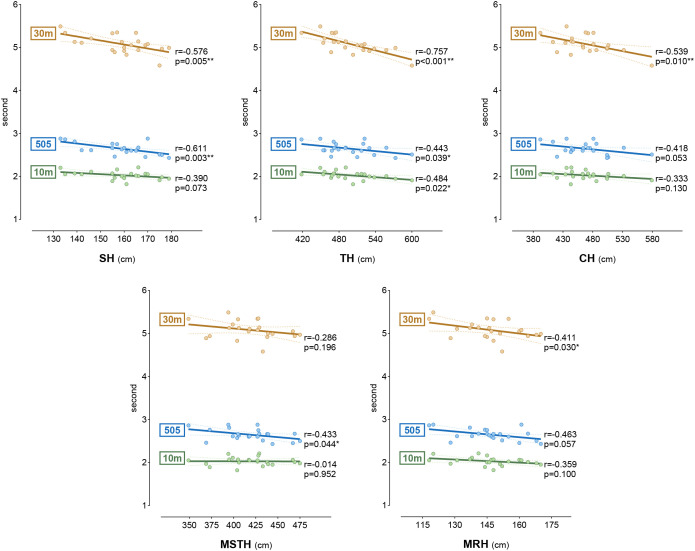
The relationship between dominant side SLHTs and sprint and agility tests.*p<0.05; **p<0.01; r results of Pearson correlation; SH, single leg hop for distance; TH, triple leg hop for distance; CH, crossover hop for distance; MSTH, medial side triple leg hop for distance; MRH, medial rotation hop for distance.

The correlation between participants’ dominant side SLHT and sprint and agility tests is presented in **Figure 5**. When the results were evaluated, there was a moderate negative correlation between the 10 m sprint and TH (r = -0.484), a moderate negative correlation between the 30 m sprint and SH and CH (r = -0.576, -0.539), a high negative correlation with TH (r = -0.757), a high negative correlation between the 505 agility and SH (r = -0.611), and a moderate negative correlation with TH, MSTH, and MRH (r = -0.443, -0.433, -0.463) (p < 0.05, p < 0.01).

**Figure 6:**
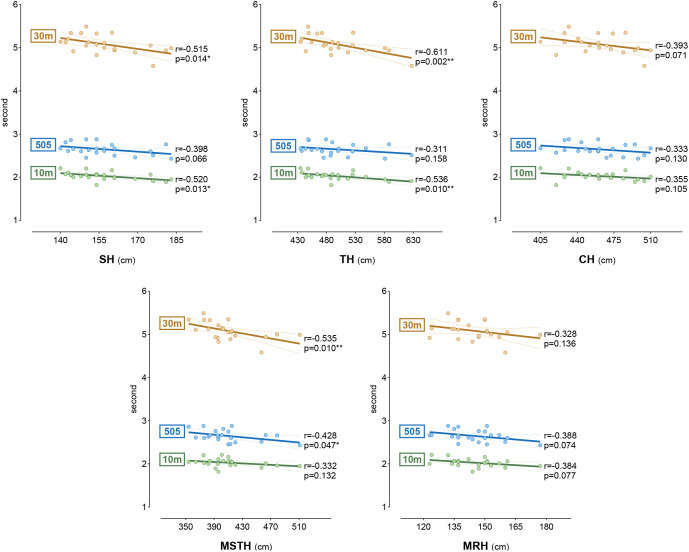
The relationship between non-dominant side SLHTs and sprint and agility tests. *p<0.05; **p<0.01; r results of Pearson correlation; SH, single leg hop for distance; TH, triple leg hop for distance; CH, crossover hop for distance; MSTH, medial side triple leg hop for distance; MRH, medial rotation hop for distance.

**Figure 6** shows the correlation between non-dominant SLHT values and the 10m sprint, 30m sprint, and 505 agility parameters. When the results were evaluated, SH was negatively correlated with the 10m and 30m sprints (r = -0.520, -0.515), TH with the 10m sprint (r = -0.536), and MSTH with the 30m sprint and 505 agility (r = -0.535, -0.428); parameters showed a moderate negative correlation (p < 0.05). A high negative correlation was found between TH and the 30 m sprint (r = -0.611) (p < 0.01).

## 4 Discussion

This cross-sectional study, conducted using randomized measurements, investigated the relationship between female volleyball players’ vertical (CMJ and DJ) and horizontal (SLHT) jump test outcomes and their sprint and agility performance. The hypothesis of the study was that there could be significant relationships between these variables, and the findings obtained supported this hypothesis. On the other hand, there was no significant difference in the jump distances between the D and ND sides in all SLHTs performed unilaterally by the participants, and the interlimb asymmetry index was within the normal range.

Although volleyball is perceived as safer than contact team sports, the incidence of injury is quite high. A study found the injury incidence leading to time lost in four-year major tournaments to be 3.8/1000 player-hours per match (95% CI 3.0-4.4) (Bere et al., 2015). In addition to acute injuries, shoulder, back, and knee injuries due to overuse are quite common among elite athletes (Timoteo et al., 2021; Skazalski et al., 2024). Therefore, developing injury prevention strategies and monitoring the prevalence of injuries among athletes is critical. A review of the literature reveals that SLHTs are used quite extensively in this area (Davies et al., 2020; Patterson et al., 2021; Latifi et al., 2024). Similarly, five different SLHTs were used in the present study. The results obtained showed that there was no significant difference between the D and ND sides in terms of jump distances in all tests and that the limb symmetry index (LSI) was within the normal range (± 10-15%) (Madsen et al., 2020; Keller et al., 2024). This result indicates functional symmetry between both sides in terms of participants’ lower extremity strength and explosive power and is a positive indicator in terms of performance optimization. Furthermore, in volleyball, where bilateral loading is quite intense, the use of both lower extremities at similar levels can be said to contribute to symmetrical motor unit activation in the neuromuscular system on both sides, balanced functioning of proprioceptive sensory receptors such as muscle spindles bilaterally, and strength production. Because similar studies conducted on healthy individuals have found that bilateral training adaptations can reduce the dominance difference and support the results of the current study (Singh et al., 2023; Kassiano et al., 2025).

On the other hand, SLHTs are not only related to injury prevalence but are also directly associated with key components of athletic performance, including explosive strength, acceleration, deceleration, agility, and speed (Aizawa et al., 2021; Hasan et al., 2021; Dominguez-Navarro et al., 2023; Yılmaz et al., 2025). One study found that increased strength and power output in the lower extremities positively affected athletes’ agility performance (Wang et al., 2025), while another study on sprinters identified significant relationships between horizontal tests (SH and TH) and sprint performance (Habibi et al., 2010). These results demonstrate the importance of the relative explosive capacity of hip and knee extensors during short-duration, high-intensity joint movements and the elastic energy stored during rapid contractions for agility and sprint performance (Zhang et al., 2021; Miller et al., 2022). The negative significant correlation between the SLHT results in the current study and the agility and sprint test results supports this thesis and contributes to the limited studies examining the relationship between sprint ability and horizontal jumps.

Vertical jump tests (CMJ, DJ, etc.) have recently attracted the attention of sports scientists and have been widely covered in the literature (Xu et al., 2024b). The metrics obtained from these test results are mostly used to correlate with critical performance parameters such as linear sprint (Barr and Nolte, 2011; Washif and Kok, 2022) and agility (Suarez-Arrones et al., 2020) in athletes. A study examining the relationship between CMJ, sprint and change of direction in an athletic population found a moderate to high correlation between jump performance and sprint and agility (Gisladottir et al., 2024). In their study on female volleyball players, Barnes et al. (2007) suggested that CMJ is an important determinant of agility performance and found that the regression analysis they obtained in their study explained approximately 34% of the variance (Barnes et al., 2007). Similarly, the current study results showed moderate to high negative correlations (r = -0.548 to -0.735) between CMJ test results and participants’ agility and sprint performance. These results showed that participants tended to decrease their sprint and agility test times in inverse proportion to the increase in CMJ height. From this result, it can be concluded that the CMJ test serves as a useful indicator of linear sprint and agility capabilities in female volleyball players. On the other hand, a similar relationship exists between the DJ test results in our study and the sprint parameters (r = -0.492 to -0.743). This result indicates that sprinting is directly related to reactive strength production and neuromuscular functions, particularly during acceleration and maximum speed phases (Markovic and Mikulic, 2010). Although team sport athletes, such as volleyball players, rely more on short-distance accelerations rather than the maximum-velocity mechanics of elite sprinters (who theoretically exhibit fast SSC ground contact times of ∼100 ms), the rapid strength production evaluated by DJ still supports their sprint capabilities (Markovic and Mikulic, 2010; Morin and Samozino, 2016). In light of this information, it can be said that the DJ test, like the CMJ test, is reliable for monitoring the sprint performance of female volleyball players. However, the same may not be true for agility, as our current results showed no significant relationship between DJ test metrics and agility performance. This shows that there is no direct link between agility and rapid SSC. From a physiological and biomechanical perspective, directional change movements require both eccentric control (braking) and concentric strength production (re-acceleration), and in this respect, they differ from DJ performance (Sheppard and Young, 2006). A similar finding was reported by Thomas et al. (2009) (Thomas et al., 2009), who found a weak correlation between DJ height and agility, noting that slow SSC function better predicted agility. Another important point is that the theoretical ground contact time during directional changes is longer than that of rapid SSC actions (∼400–600 ms). Therefore, agility skills are more relevant to slow- and medium-speed SSC functions. Good fast SSC function alone is not sufficient for an athlete, and components such as slow SSC, balance, and technique determine success in agility (Morin and Samozino, 2016).

### 4.1 Limitations

While the current study has its strengths, it also has certain limitations that should be taken into account. Jump, sprint, and agility performances are influenced by multiple factors, including muscle fiber type distribution, tendon stiffness, and neuromuscular coordination. However, the relevant parameters are not included in this study, and this limits the full explanation of the physiological mechanisms underlying the correlation analyses obtained. Finally, the relatively small sample size in the study, consisting solely of female volleyball players and belonging to the same performance level, made it difficult to directly generalize the findings to volleyball athletes. Therefore, diversifying and expanding the sample group in future studies will contribute to more detailed analyses and the generalizability of the findings.

## 5 Conclusions

This study revealed moderate to high correlations between lower extremity vertical (CMJ-DJ) and horizontal (SLHT) jump parameters and sprint and agility performance. The results indicate that vertical and horizontal neuromuscular and functional strength production mechanisms play a critical role in determining linear acceleration and directional change performance in female volleyball players. Also, it has been determined that the contribution of reactive strength metrics to linear acceleration performance is more pronounced compared to agility. In light of this information, including plyometric exercises in training plans that develop not only vertical but also horizontal strength production can significantly improve sprint and agility performance. In further studies, in addition to vertical lower-extremity jump tests that allow the assessment of neuromuscular performance, evaluating horizontal functional tests and their relationship with performance parameters is important for a more comprehensive assessment of results.

## Data Availability

The raw data supporting the conclusions of this article will be made available by the authors, without undue reservation.
